# Etiologic Agents of Central Nervous System Infections among Febrile Hospitalized Patients in the Country of Georgia

**DOI:** 10.1371/journal.pone.0111393

**Published:** 2014-11-04

**Authors:** Tamar Akhvlediani, Christian T. Bautista, Roman Shakarishvili, Tengiz Tsertsvadze, Paata Imnadze, Nana Tatishvili, Tamar Davitashvili, Tamar Samkharadze, Rusudan Chlikadze, Natia Dvali, Lela Dzigua, Mariam Karchava, Lana Gatserelia, Nino Macharashvili, Nana Kvirkvelia, Engy Emil Habashy, Margaret Farrell, Emily Rowlinson, James Sejvar, Matthew Hepburn, Guillermo Pimentel, Erica Dueger, Brent House, Robert Rivard

**Affiliations:** 1 I. Javakhishvili Tbilisi State University, Department of Neurology and Neurosurgery, Tbilisi, Georgia; 2 US Army Medical Research Unit-Georgia (USAMRU-G), Tbilisi, Georgia; 3 Walter Reed Army Institute of Research, Silver Spring, Maryland, United States of America; 4 P. Sarajishvili Institute of Clinical Neurology and Neurosurgery, Tbilisi, Georgia; 5 Scientific Research Center of Infectious Pathology, AIDS, and Clinical Immunology, Tbilisi, Georgia; 6 National Center for Disease Control and Public Health, Tbilisi, Georgia; 7 Neurology Department of the Iashvili Children's Hospital, Tbilisi, Georgia; 8 Global Disease Detection and Response Program, U.S. Naval Medical Research Unit No. 3, Cairo, Egypt; 9 National Center for Emerging and Zoonotic Infectious Diseases, Centers for Disease Control and Prevention, Atlanta, Georgia, United States of America; 10 National Center for Immunization and Respiratory Diseases, Centers for Disease Control and Prevention, Atlanta, Georgia, United States of America; 11 US Army Medical Research Institute of Infectious Diseases, Frederick, Maryland, United States of America; University of Cincinnati School of Medicine, United States of America

## Abstract

**Objectives:**

There is a large spectrum of viral, bacterial, fungal, and prion pathogens that cause central nervous system (CNS) infections. As such, identification of the etiological agent requires multiple laboratory tests and accurate diagnosis requires clinical and epidemiological information. This hospital-based study aimed to determine the main causes of acute meningitis and encephalitis and enhance laboratory capacity for CNS infection diagnosis.

**Methods:**

Children and adults patients clinically diagnosed with meningitis or encephalitis were enrolled at four reference health centers. Cerebrospinal fluid (CSF) was collected for bacterial culture, and in-house and multiplex RT-PCR testing was conducted for herpes simplex virus (HSV) types 1 and 2, mumps virus, enterovirus, varicella zoster virus (VZV), *Streptococcus pneumoniae*, HiB and *Neisseria meningitidis*.

**Results:**

Out of 140 enrolled patients, the mean age was 23.9 years, and 58% were children. Bacterial or viral etiologies were determined in 51% of patients. Five *Streptococcus pneumoniae* cultures were isolated from CSF. Based on in-house PCR analysis, 25 patients were positive for *S. pneumoniae*, 6 for *N. meningitidis*, and 1 for *H. influenzae*. Viral multiplex PCR identified infections with enterovirus (n = 26), VZV (n = 4), and HSV-1 (n = 2). No patient was positive for mumps or HSV-2.

**Conclusions:**

Study findings indicate that *S. pneumoniae* and enteroviruses are the main etiologies in this patient cohort. The utility of molecular diagnostics for pathogen identification combined with the knowledge provided by the investigation may improve health outcomes of CNS infection cases in Georgia.

## Introduction

Central nervous system (CNS) infections continue to afflict populations worldwide, especially due to their associations with mortality and long-term disability [1]. In spite of the harm caused by these infections, they remain poorly described in many regions of the world. Most bacterial meningitis (BM) cases are attributable to *Haemophilus influenzae* type B (HiB), *Streptococcus pneumoniae or Neisseria meningitidis*. The etiologic distribution of these infections varies with geographic location and age group. HiB tends to have a higher prevalence in infants and children less than five years of age [2]. In contrast, *S. pneumoniae* and *N. meningitidis* are responsible for most bacterial cases in adults [3], [4]. The introduction of vaccines for these pathogens has influenced the epidemiology of BM in high-income countries, particularly the incidence of HiB-related meningitis [5]. Currently, *S. pneumoniae* and *N. meningitidis* are the two leading causes of BM in North America and Western Europe [6]. Developing countries and countries with economies in transition have historically been reluctant to introduce HiB or other BM vaccines, primarily due to the high cost of vaccines and insufficient information regarding the burden of CNS infection associated with different bacterial pathogens [7], [8].

Viral CNS infections are important causes of neurologic illness, and may result in meningitis, encephalitis, and myelitis with varying degrees of severity [9]. The most commonly identified viruses leading to CNS infections are enteroviruses, herpesviruses, and arboviruses. The determination of a definitive etiology in suspected viral CNS infections is challenging. In most cases, the etiology is unknown, even after extensive diagnostic testing, however, this situation is improving due to availability of newer molecular diagnostics techniques [10]–[12].

In the country of Georgia, limited research data suggest that three bacterial pathogens (HiB, *S. pneumoniae, and N. meningitidis*) are the main causes of BM [13]. Old data from other former Soviet Republics indicate that before the introduction of the HiB vaccine, 50% of childhood BM in Russia was due to HiB [14]. Due to the unavailability of reliable, affordable laboratory diagnostic tests in Georgia, there are few confirmed diagnoses of viral CNS infections. Consequently, the true incidence and etiology of bacterial and viral meningitis are not well described. Physicians routinely rely upon clinical assessment and their own medical judgment and experience to differentiate between BM and viral meningitis (VM), and patients are treated empirically; these clinical assessments may frequently lead to misdiagnosis. Empiric therapy may lead to inappropriate and excessive use of antibiotics and promote antibiotic resistance.

The aim of this study was to characterize the spectrum of CNS infection pathogens in febrile hospitalized patients, utilizing both traditional culture techniques and molecular diagnostics to improve the laboratory diagnostic assessment of these patients. This study describes laboratory findings associated with *S. pneumoniae*, *N. meningitidis*, HiB, enterovirus, varicella zoster virus (VZV), herpes simplex virus type 1 (HSV-1), herpes simplex virus type 2 (HSV-2), and mumps infections, provides detailed clinical information about these cases, and analyzes the relationships between clinical findings and laboratory results.

## Methods

### Study Design

This hospital-based study was conducted between October 2010 and May 2012 at four reference clinics in Tbilisi, the capital of Georgia: 1) Scientific Research Center of Infectious Pathology, AIDS, and Clinical Immunology; 2) P. Sarajishvili Clinical Neurology and Neurosurgery Institute; 3) Iashvili Children's Central Hospital; and 4) the Neurology Department of S. Khechinashvili University Clinic. Patients were eligible for the study if they had: 1) a current or history of self-reported or documented fever of ≥38C° during the course of the illness; 2) one or more signs of meningitis and/or encephalitis (severe headache, photophobia, nausea/vomiting, meningeal signs, petechial/purpural rash, altered mental status, seizures, and lethargy); and 3) clinical indication for lumbar puncture (LP), as determined by the attending physician. Exclusion criteria for enrollment included: 1) patient with a documented non-infectious cause for neurologic illness; 2) patient less than two months of age; or 3) a post-neurosurgical, trauma, or immune-compromised patient. Trained study site physicians at each study site reviewed hospital admission logs on a daily basis to identify patients with a diagnosis of acute meningitis or encephalitis. Patients or their legal guardians provided written informed consent prior to enrollment. The study was approved by the institutional review boards of the National Center for Disease Control and Public Health in Georgia, the U.S. Army Medical Research Institute of Infectious Diseases, the Walter Reed Army Institute of Research, and Naval Medical Research Unit-3 in Cairo, Egypt. The medical care of patients was conducted according to routine local clinical practice and was not influenced by the study.

Information on demographics, risk factors, clinical features, and epidemiologic factors were collected on a standardized report form by the study site physicians. Data on medical history, laboratory tests performed during the care of the patient and treatment rendered was also collected on this form. In Georgia, LP is standard practice for patients with a suspected CNS infection. For study purposes, participants were asked to consent to the collection of an additional 1–3 ml of cerebrospinal fluid (CSF) beyond the volume normally drawn for examination and diagnosis (3–5 ml). CSF was preferentially collected prior to antibiotic treatment, consistent with the patient's condition and physician treatment. The level of consciousness was assessed using the Glasgow Coma Scale [15]. In patients with suspected brain hemorrhage or a focal brain lesion, computed tomography (CT) or magnetic resonance imaging (MRI) scan was conducted as part of their routine medical assessment.

### CSF Laboratory Testing

The CSF laboratory testing panel included cell counts and differential, glucose and protein levels. Based on medical history, clinical presentation, and CSF parameters, clinical suspected diagnoses were made by treating clinicians, and patients were classified into cases with BM, VM, tuberculosis (TB) meningitis, or viral encephalitis (VE) [16]. As part of the research study, several laboratory tests were performed for all patients, regardless of clinical diagnoses. A CSF gram stain was performed; an aliquot was plated on chocolate agar and incubated at 37°C in CO_2_ for 72 hours. In-house singleplex PCR was performed to detect HiB, *N. meningitidis*, and *S. pneumoniae*. A multiplex PCR test (Fast-Track Diagnostics (FTD) Viral Meningitis for ABI 7500 and Rotor-Gene 6000) was used to detect HSV-1, HSV-2, mumps, enterovirus, and VZV. MagNA pure automatic extractor, which is a closed system, was used to minimize contamination. Every run contained 30 samples +1 human sample control (HSC) +1 NTC (water). The HSC ensured that the extraction was done properly by doing the RNP gene and the water to ensure there was no contamination during extraction. The bacterial real time PCR was singleplex, where each target was done in a separate PCR plate with no primers contamination or mix up. The specimens were batched for PCR testing; results were reported back to the treating physicians on a periodic basis, but were not intended to guide clinical management. Confirmatory testing for TB meningitis was not part of this study. All suspected TB meningitis cases were referred to the National Center for Tuberculosis and Lung Diseases for care.

### Statistical Analysis

Chi-square test was used to compare percentages and the Mann-Whitney U test to compare means. Odds ratios (OR) with 95% confidence intervals were estimated to measure the association between selected covariates and clinical diagnosis, and were then adjusted (AOR) for age in years, gender, and study site using logistic regression analysis. All p-values were two sided and the significance level was set at 0.05. All analyses were performed using Epi-Info version 7 and SPSS software.

## Results

### Demographics

A total of 140 patients were enrolled, and of these, complete clinical information was available in 134 (96%). The mean age was 23.9 years and 58% of patients were children less than 18 years of age. Over 51% of participants were male, 67% were urban residents, including 40% who were residents of the capital city of Tbilisi (Table 1). Among study participants, 33% reported having had contact with animals within two weeks prior to enrollment. Specific animals reported were: dogs (28%), poultry (25%), cats (16%), cattle (12%), swine (6%), sheep/goats (4%), and rodents (4%). Regarding agricultural activities, 10% of patients reported participating in soil cultivation, harvesting, cutting grass, etc. Travel outside Georgia during the month prior to illness was uncommon (<1%).

**Table 1:** Baseline characteristics of 140 patients presenting with febrile acute central neurologic system infections, country of Georgia.

Feature	no (%)
Age (years), mean (SD)	23.9 (22.8)
Male	71 (53)
Tbilisi residence	53 (40)
Iashvili Chidren's Hospital	47 (35)
Animal contact	44 (33)
Agriculture activity	14 (10)
Tick bite	1 (0.7)
Mosquito bite	25 (19)
Travel outside Georgia	1 (0.7)

Note: SD, standard deviation.

### Clinical and Laboratory Data

Thirty-one percent of patients had been hospitalized elsewhere for illnesses related to their CNS illness during the month before presenting to one of the study sites. Over 33% of participants received antibiotics before hospital admission, 30% were referred from other health centers, and 15% had a pre-existing medical condition. In addition, 16% of patients reported having had symptoms suggestive of another infection in the month before they developed CNS illness (43% of these patients had a middle ear infection). At hospital admission, the median temperature was 38°C (range  = 37.5–40°C). Patients were symptomatic for a mean duration of 4.4 days (standard deviation  = 6.3) before presenting to the hospital. The most common signs and symptoms reported were fever, headache, nausea/vomiting, and depressed mood, and to a lesser extent, rash, seizures, abdominal pain, diarrhea, pain in joints, and skin lesions (Table 2).

**Table 2:** Frequency of signs and symptoms among 140 patients presening with acute febrile central nervous system syndrome, country of Georgia.

Signs/symptoms	no (%)
Fever	138 (98)
Headache	128 (91)
Nausea/vomiting	125 (90)
Depressed mood	114 (81)
Stiff neck	110 (79)
Pain behind the eyes	96 (69)
Chills	82 (59)
Photophobia	75 (54)
Night sweats	60 (43)
Sleep disturbance	63 (45)
Cough	28 (20)
Shaking/rigors	24 (17)
Personality change	21 (15)
Sore throat	20 (14)
Rash	10 (8)
Seizure	10 (7)
Abdominal pain	8 (6)
Muscle soreness	7 (5)
Pain in joints	7 (5)
Diarrhea	5 (4)
Skin lesions	2 (1)
Unusual bleeding	0 (0)

The percentage of patients who reported having received Bacillus Calmette-Guerin (BCG), measles/mumps/rubella (MMR), and varicella vaccines were 73%, 53%, and 0%, respectively. Only one patient remembered receiving HiB vaccine and one patient had received the *S. pneumoniae* vaccine.

Based on the Glasgow Coma Scale (GCS), three patients were in deep coma (<5) at time of admission, and seven patients had a GCS score between 6 and 10. Kernig's and Brudzinski's signs were seen in 50% and 40% of patients, respectively. An altered mental status (confusion) was observed in 22% of patients. Cranial nerve palsy was detected in 7% of patients, with the most common finding being peripheral facial nerve weakness (70%). Sensory deficit was uncommon (2%) and focal motor deficit was detected in 8% of patients.

CSF was clear in 81 (58%) patients and turbid in 45 (32%) patients. Nine (7%) CSF specimens were considered traumatic taps and had gross blood contamination. CSF was unavailable on five patients. The median values of CSF parameters for patients with BM were: white blood cells (WBC) count 1,530 cells/mm^3^ (range: 53–100,000 cells/mm^3^), neutrophils 78% (range: 20–95%), lymphocytes 22% (range: 5–80%), protein 66 mg/dl (range: 0.17–660 mg/dl), and glucose 44 mg/dl (range: 0–167 mg/dl). These and additional CSF parameters are presented in Table 3.

**Table 3:** Median Values and Ranges of Cereprospinal Fluid (CSF) Parameters among patients with clinically diagnosed central nervous system infections, country of Georgia.

CSF Parameter	Bacterial Meningitis	Viral Meningitis	Tuberculosis Meningitis	Encephalitis
	n = 75	n = 39	n = 8	n = 12
White Blood Cells (WBC) (cells/mm3)	1,530 (53–100,000)	71 (12–450)	465 (65–3500)	29 (7–133)
Neutrophils (%)	78 (20–95)	57 (0–71)	62 (0–68)	46 (30–58)
Lymphocytes (%)	22 (5–80)	42 (12–100)	39.5 (32–100)	54 (42–70)
Protein (mg/dl)	66 (0.17–660)	11.6 (0–66)	66 (33–660)	33 (0.03–330)
Glucose (mg/dl)	44 (0–167)	69 (31–158)	39 (22–145.8)	71 (37–147)

CT and MRI results were abnormal in 11 of 35 (31%) and 14 of 31 (45%) of patients, respectfully. Abnormal findings included sinusitis, ethmoidal osteoma, mastoiditis, multiple brain abscesses, and subarachnoid cyst. Over 62% of patients received antibiotics during their hospital stay, most commonly ceftriaxone and vancomycin.

### Clinical Diagnosis

Based on clinical diagnosis, 75 (56%) patients were classified by treating clinicians as BM, 39 (29%) as VM, 8 (6%) as TB meningitis and 12 (9%) as encephalitis (Table 3). In four patients, this was the second diagnosis of BM during their lifetime.

There were no differences between clinically diagnosed BM, VM, TB meningitis, and encephalitis by gender, Tbilisi residence, urban residence, animal contact, another illness in the month before patient developed neurologic signs, or hospitalization in the month prior to study enrollment. BM cases had a significantly higher mean age (28.8 years) and duration of illness (4.8 days) compared to their counterparts, while encephalitis cases reported significantly more involvement in agricultural activity (33%).

Patients clinically diagnosed with BM or TB meningitis had a significantly higher percentage of Kernig's sign and signs/symptoms of chills and sleep disturbances compared to VM and encephalitis cases (Table 4). The hospitalization duration was significantly longer among BM and encephalitis cases than among patients diagnosed with VM or TB meningitis. Changes in personality were more likely to occur among BM cases, followed by encephalitis and TB meningitis cases. CSF was turbid in 58% (n = 43) of the BM cases, 3% (n = 1) of VM cases, but clear among all encephalitis and TB meningitis cases. Most VM and BM cases had a complete recovery by the time of hospital discharge and 60% of encephalitis and TB meningitis cases left the hospital with improved condition but still with some sequelae. Median duration of hospitalization for BM was 22 days (range: 3–152 days); for VM 9.5 days (range: 2–43 days);, and for encephalitis 10 days (range: 3–22 days) TB meningitis cases were unable to be followed up prospectively.

**Table 4:** Selected characteristics of the study patients with acute central nervous system infections classified by clinical diagnosis, country of Georgia.

Feature	Bacterial Meningitis (n = 75)	Viral Meningitis (n = 39)	Tuberculosis Meningitis (n = 8)	Encephalitis (n = 12)	*P-value*
Age (years), mean (SD)	28.8 (24.8)	13.5 (16.8)	40.4 (19.6)	21.5 (15.7)	0.004
Male	39 (52)	19 (50)	5 (62)	8 (67)	0.717
Tbilisi residence	29 (39)	17 (44)	4 (50)	3 (25)	0.633
Urban residence	52 (69)	28 (72)	4 (50)	4 (33)	0.057
Animal contact	21 (28)	14 (36)	3 (37)	6 (50)	0.450
Agriculture activity	6 (8)	2 (5)	2 (25)	4 (33)	0.017
Duration of illness (days), mean (SD)	4.8 (7.8)	2.4 (2.2)	3.6 (2.1)	8.2 (4.8)	<0.001
Another infection in the prior month	16 (21)	2 (5)	0 (0)	3 (25)	0.067
Hospitalized in the prior month	27 (36)	6 (15)	4 (50)	5 (42)	0.064
Days of hospitalization, mean (SD)	14.5 (6.2)	9.3 (4.1)	11.7 (8.5)	17.3 (12.3)	<0.001
Kernig's sign	45 (60)	14 (37)	6 (75)	2 (17)	0.005
Brudzinski's sign	31 (41)	17 (46)	4 (50)	2 (17)	0.310
Altered state of consciousness	21 (28)	3 (8)	2 (25)	4 (33)	0.090
Severe or moderate disability	10 (14)	1 (3)	1 (12)	2 (17)	0.289
Motor deficit	3 (4)	3 (8)	0 (0)	5 (45)	<0.001
Chillls	50 (70)	23 (60)	4 (50)	2 (18)	0.009
Change of personality	17 (24)	1 (3)	1 (12)	2 (18)	0.040
Sleep disturbance	42 (59)	10 (26)	4 (50)	3 (27)	0.006
Cranial nerve palsy	2 (3)	2 (5)	2 (25)	4 (36)	<0.001
Turbid CSF	43 (58)	1 (3)	1 (12)	0 (0)	<0.001
Illness resolved at hospital discharge	62 (87)	37 (95)	2 (25)	3 (25)	<0.001

Note: SD, standard deviation; CSF, cerebrospinal fluid.

Five patients with BM presented with petechial rash. In one patient with VZV-associated encephalitis, a papular rash was detected. One 12-year old female diagnosed with encephalitis reported a prior tick bite. Of 25 cases positive for *S. pneumoniae* by PCR, 19 patients had a full recovery (76%), four were discharged with some residual symptoms (16%); one patient, a 74– year old male with pre-existing medical conditions died, the only fatal case in the study. The outcome was unknown in one case of *S. pneumoniae*. Of six cases positive for *N. meningitidis* by PCR, 5 (83%) had a full recovery and one (17%) was discharged with some residual symptoms. One PCR-positive case of HiB was discharged with residual symptoms.

Significant risk factors associated with BM when compared with VM were: age (OR for each year of age  = 1.04, 95% CI = 1.01–1.07), duration of illness per day (OR = 1.21, 95% CI = 1.02–1.44), hospitalization in the past month for another illness (OR = 4.10, 95% CI = 1.28–13.19), sleep disturbances (OR = 4.04, 95% CI = 1.58–10.30), and presence of Kernig's sign (OR = 4.04, 95% CI = 1.20–13.61).

### CSF and PCR Laboratory Analysis

Table 5 shows the distribution of laboratory results within each clinical group. Bacterial or viral etiology diagnosis was made in 51% of patients. Five *S.pneumoniae* cultures were positive from CSF. Based on in-house PCR analysis, 25 patients were positive for *S. pneumoniae*, six for *N. meningitidis*, and one for HiB. The *H. influenzae* case was also positive for *S. pneumoniae* and one patient was positive for both *S. pneumoniae* and *N. meningitis*. The viral multiplex PCR analysis indicated that 26 patients were infected with enterovirus, four patients with VZV, and two patients with HSV-1. No patient was positive for mumps or HSV-2. In one case, both enterovirus and *S. pneumoniae* PCR tests were positive. *S. pneumoniae* and enterovirus were the most commonly detected etiologies of BM and VM cases.

**Table 5:** Laboratory results for patients diagnosed with bacterial meningitis, viral meningitis, TB meningitis, and encephalitis, country of Georgia.

Laboratory result	Bacterial Meningitis	Viral Meningitis	Tuberculosis Meningitis	Encephalitis
	n = 75	n = 39	n = 8	n = 12
*S. pneumoniae* *	5	0	0	0
*S. pneumoniae* **	20	1	3	0
*N. meningitidis*	5	0	1	0
*H. influenzae*	1	0	0	0
Enterovirus	10	16	0	0
Varicella zoster virus	0	3	0	1
Herpes simplex virus type 1	1	0	0	1
Herpes simplex virus type 2	0	0	0	0
Mumps	0	0	0	0

*By cerebrospinal fluid culture.

**By in-house singleplex PCR analysis.

## Discussion

This hospital-based study provides information about the main pathogens causing CNS infections in febrile hospitalized patients in Georgia. Our data suggest that the most common bacterial pathogen causing meningitis in Georgia is *S. pneumoniae* and that the most common viral pathogens are enteroviruses. The observation that bacterial culture was positive in only five cases (3.6%) may be attributed to antibiotic use prior to hospital admission. These findings highlight the utility of molecular diagnostics for improving pathogen identification for CNS infections. While the use of molecular diagnostic methods substantially enhanced pathogen detection in this study, causative agents were still only detected in half of patients. This low percentage was similar to that reported in other studies [12], [17], and is likely a consequence of previous antibiotic use, clearance of virus resulting in absence of detectable nucleic acid, or infection with pathogens not included in the diagnostic panel for our study.

One of the aims of the study was to compare clinical diagnosis with laboratory-based pathogen identification. We noted clear discrepancies between clinical diagnosis and laboratory identification in our study, most notably for VM. Specifically, ten cases of enterovirus meningitis were erroneously classified as BM which led to unnecessary use of antibiotics. This important finding supports the use of molecular diagnostics in this group of cases, when feasible and affordable, as accurate identification of VM could assist in the targeted use of antibiotics and appropriate allocation of hospital resources. In addition, our study highlights the need for developing a standardized clinical case definition for BM, viral VM, and encephalitis that are evidence-based and correlate to pathologic diagnosis. Such case definitions would allow for comparability of findings across studies, and lead to a more accurate clinical interpretation/diagnosis at the bedside, allowing for better empiric management.

Another aim of our study was to assess the impact of HiB vaccination on the etiology of CNS infections in Georgia. Ten months before the study was initiated, a HiB vaccination campaign was initiated in Georgia. However, since very few of our patients reported being vaccinated with HiB vaccine, and we identified only one case of HiB meningitis, we were unable to adequately assess this. *S. pneumoniae* was the predominant bacterial pathogen (25 positive cases); given the fact that Georgia is anticipating the introduction of *S. pneumoniae* vaccine in the near future, our study may provide valuable baseline data to assess the efficacy of this vaccine. Further studies are warranted to discern the long-term impact of HiB and *S. pneumoniae* vaccines in the Georgian population.

A defined causative agent was identified in only two cases of encephalitis (one case of VZV and one case of HSV-1). This is consistent with prior etiologic studies of encephalitis of presumed infectious origin in which, despite extensive testing, a causal agent was identified in only 30 to 50% of cases [18]. The employment of molecular diagnostics for encephalitis is helpful, but still results in a lack of definitive diagnosis in the majority of cases. This investigation highlights the need for better diagnostic strategies for this syndrome as well as enhanced understanding of non-infectious causes of encephalitis, such as immune-mediated encephalitides, as well as the potential for new, currently unrecognized pathogens as causes of encephalitis.

There were several cases of apparent co-infection detected among the study patients. To date, there has not been any reported association between HiB and *S. pneumoniae* meningitis, however, a case of severe pneumonia caused by the co-infection of these two pathogens was recently described by Akuzawa et al. [19]. Increase in the use of molecular diagnostics may lead to more frequent detection of co-infection in CNS cases, although it should be noted that a false positive result on one or both of the pathogens detected in our cases based upon our in-house assay cannot be entirely excluded. Co-infection in CSN patients will be an important topic to assess with further study in larger cohorts and with subsequent validation of results.

It is well known that *S.pneumoniae* meningitis may be associated with severe sequelae. Even in developed countries, the percentage of sequelae is up to 31% [20]. In this study, all but three of 25 patients (78%) were discharged with complete recovery. Potential explanations of this observation include early and widespread utilization of antibiotics (especially antibiotics with good CNS penetration) and molecular diagnosis of cases with lower concentrations of bacteria in the CNS. To test these hypotheses, a comparison of the severity of infection between culture-positive and PCR-positive cases of BM in larger cohort studies is needed.

Our study had some limitations. First, the generalizability of our research findings is limited. Since our study sites represent all of the reference clinics in Georgia (except for the National Center for Tuberculosis and Lung Diseases, to which TB meningitis patients are referred), we can make some general assumptions regarding CNS infection in the country; however, extrapolation to other geographic areas outside of the South Caucasus region are probably not possible, given the geographic diversity of pathogens causing CNS infections, worldwide [21]. Second, the number of specific pathogens for laboratory testing was limited and there are other possible etiologies of CNS infection in Georgia (e.g., tick-borne encephalitis virus and measles virus), but were not assessed in our study. Lastly, the biological samples associated with the study were limited to CSF, which may be problematic for diagnosis since pathogens may be present in the CNS transiently. For many agents of CNS infections (e.g., arboviruses), serologic studies to detect the presence of virus-specific IgM antibodies in serum or a rise in antibody titer can be useful; this study did not include serologic assays.

In conclusion, this study provides valuable baseline data regarding the frequency and etiology of CNS infections in Georgia, and can serve as a foundation for future assessment of the impact of the introduction of new diagnostic platforms, therapies, and vaccines in Georgia. Many of our findings substantiate the results of prior investigations regarding pathogens causing CNS infections, and the challenges of arriving at a laboratory-confirmed diagnosis. However, our study was the first of its kind in the South Caucasus, and further characterized the nature of pathogens causing CNS infections in the region, facilitated by the employment of molecular diagnostics. Observations such as the clinical misclassification of many enteroviral meningitis cases as bacterial infections demonstrate the utility of these assays. Enhanced understanding of the key characteristics of CNS infections in Georgia will assist in public health and health care planning, as interventions strategies are considered and eventually may translate into improved health outcomes in Georgia.
